# Carbon flux around leaf-cytosolic glyceraldehyde-3-phosphate dehydrogenase introduces a ^13^C signal in plant glucose

**DOI:** 10.1093/jxb/erab316

**Published:** 2021-07-05

**Authors:** Thomas Wieloch, Roland Anton Werner, Jürgen Schleucher

**Affiliations:** 1 Department of Medical Biochemistry and Biophysics, Umeå University, 901 87 Umeå, Sweden; 2 Department of Environmental Systems Science, ETH Zürich, Universitätstrasse 2, 8092 Zürich, Switzerland; 3 MPI of Molecular Plant Physiology, Germany

**Keywords:** Carbon allocation, carbon flux, carbon stable isotopes, cytosolic oxidation–reduction cycle, energy metabolism, glyceraldehyde-3-phosphate dehydrogenase, intramolecular isotope analysis, isotope fractionation model, primary carbon metabolism

## Abstract

Within the plant and Earth sciences, stable isotope analysis is a versatile tool conveying information (*inter alia*) about plant physiological and paleoclimate variability across scales. Here, we identify a ^13^C signal (i.e. systematic ^13^C/^12^C variation) at tree-ring glucose C-4 and report an experimentally testable theory on its origin. We propose the signal is introduced by glyceraldehyde-3-phosphate dehydrogenases in the cytosol of leaves. It conveys two kinds of (potentially convoluted) information: (i) commitment of glyceraldehyde 3-phosphate to 3-phosphoglycerate versus fructose 1,6-bisphosphate metabolism; and (ii) the contribution of non-phosphorylating versus phosphorylating glyceraldehyde-3-phosphate dehydrogenase to catalysing the glyceraldehyde 3-phosphate to 3-phosphoglycerate forward reaction of glycolysis. The theory is supported by ^13^C fractionation modelling. Modelling results provide the first evidence in support of the cytosolic oxidation–reduction (COR) cycle, a carbon-neutral mechanism supplying NADPH at the expense of ATP and NADH, which may help to maintain leaf-cytosolic redox balances. In line with expectations related to COR cycling, we found a positive correlation between air vapour pressure deficit and ^13^C discrimination at glucose C-4. Overall, ^13^C-4 signal analysis may enable an improved understanding of leaf carbon and energy metabolism.

## Teaser

A ^13^C signal at plant glucose C-4 provides information about carbon flux around leaf-cytosolic glyceraldehyde-3-phosphate dehydrogenases and associated energy metabolism. Isotope fractionation modelling provides first evidence of the cytosolic oxidation–reduction cycle.

## Introduction

Relative abundances of stable carbon isotopes (^13^C/^12^C ratios) in plant metabolites convey information about physiological processes and associated environmental controls (Farquhar *et al.*, 1982; McCarroll and Loader, 2004; Schmidt *et al.*, 2015). Within the plant and Earth sciences, measurements are conventionally performed on whole metabolites which gives access to a single ^13^C signal (the term ^13^C signal denotes systematic ^13^C/^12^C variation). This signal has evolved into a standard tool for retrospective assessments of plant carbon uptake and associated properties, such as photosynthetic water-use efficiency (Farquhar *et al.*, 1982).

However, in biochemistry, pronounced ^13^C differences among individual carbon positions of plant metabolites have long been established (Abelson and Hoering, 1961). Recently, a time series analysis on plant glucose revealed three novel ^13^C signals at the intramolecular level (Wieloch *et al.*, 2018). Such signals are introduced by enzyme reactions downstream of Rubisco (Schmidt *et al.*, 2015; Wieloch *et al.*, 2018). For instance, ^13^C signals at glucose C-1 and C-2 were attributed to shifts of the phosphoglucose isomerase reaction from kinetic to equilibrium conditions (EC 5.3.1.9; Wieloch *et al.*, 2018). Hence, it is now clear that the conventional whole-molecule approach averages information about distinct ecophysiological processes, a fundamental limitation. In contrast, the intramolecular approach resolves this information and may enable assessments not only of carbon uptake at higher quality but also of carbon allocation and associated environmental controls. It opens a new door to metabolic and environmental information.

Manipulation and monitoring experiments can cover short to medium time scales. In contrast, ^13^C signals enable retrospective analyses of physiological processes and their environmental controls on time scales from hours to millennia (Farquhar *et al.*, 1982; McCarroll and Loader, 2004; Schmidt *et al.*, 2015). Retrieving ^13^C signals from archived plant materials (e.g. tree rings) may enable elucidation of long-term properties, such as plant acclimation, and climate trends.

**Fig. 1.**
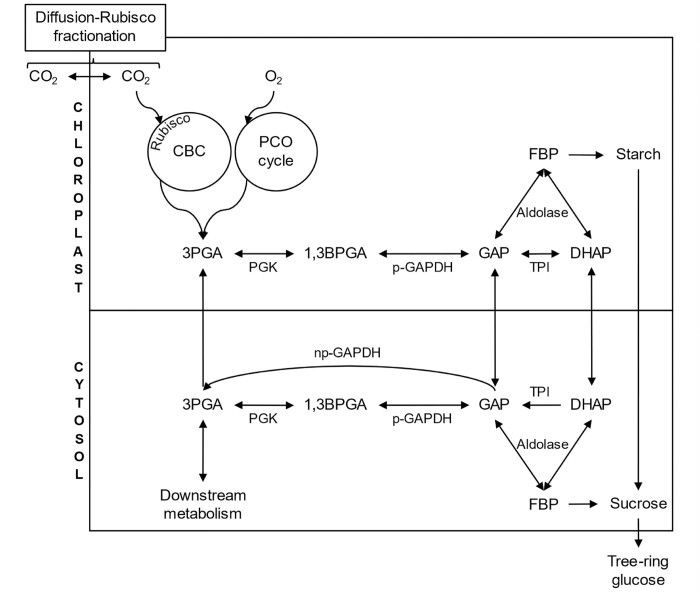
Carbon metabolism in plant leaves. In our ^13^C discrimination model, cytosolic GAP has two fates: (i) glycolytic 3PGA and downstream metabolism, and (ii) FBP and downstream metabolism including the biosynthesis of tree-ring glucose (Fig. 4). Forward conversion of GAP to 3PGA can proceed via either non-reversible np-GAPDH or reversible p-GAPDH and PGK, while the reverse conversion of 3PGA to GAP relies on PGK and p-GAPDH. Both the commitment of GAP to 3PGA versus FBP metabolism and the contribution of np-GAPDH versus p-GAPDH to catalysing the GAP to 3PGA forward conversion may alter the ^13^C/^12^C ratio at tree-ring glucose C-4 and respond to environmental cues. *In vivo*, the reaction catalysed by cytosolic TPI is strongly displaced from equilibrium on the side of GAP (Wieloch *et al.*, 2021, Preprint). That is, GAP and DHAP are not in isotopic equilibrium because cytosolic flux from GAP to DHAP is negligible. Parts of the PCO cycle reside outside chloroplasts, in peroxisomes, and mitochondria. Figure 2 shows the depicted reactions in detail. Abbreviations: 1,3BPGA, 1,3-bisphosphoglycerate; 3PGA, 3-phosphoglycerate; Aldolase, fructose-bisphosphate aldolase; CBC, Calvin–Benson cycle; DHAP, dihydroxyacetone phosphate; FBP, fructose 1,6-bisphosphate; GAP, glyceraldehyde 3-phosphate; np-GAPDH, non-phosphorylating glyceraldehyde-3-phosphate dehydrogenase; PCO cycle, photosynthetic carbon oxidation (photorespiration) cycle; p-GAPDH, phosphorylating glyceraldehyde-3-phosphate dehydrogenase; PGK, phosphoglycerate kinase; TPI, triose-phosphate isomerase.

**Fig. 2.**
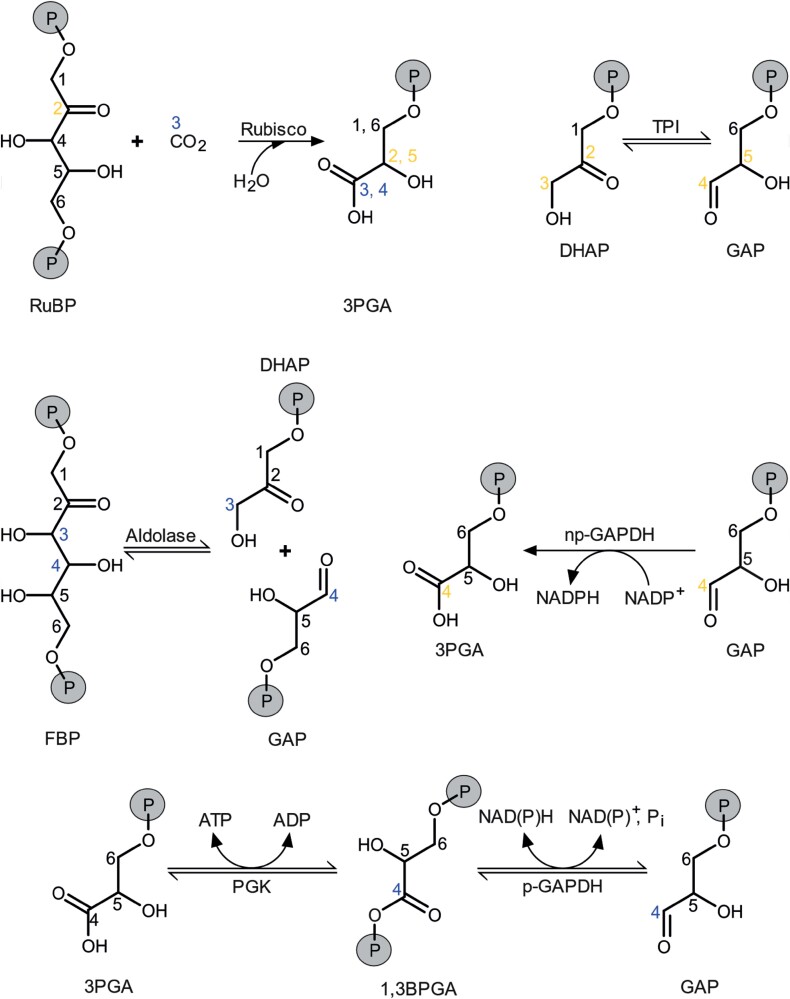
Reactions associated with carbon isotope effects. Carbon numbering according to related carbon positions in glucose. Blue: carbon bond modifications reportedly accompanied by primary ^13^C isotope effects. Orange: carbon bond modifications possibly accompanied by primary ^13^C isotope effects. Abbreviations: 1,3BPGA, 1,3-bisphosphoglycerate; 3PGA, 3-phosphoglycerate; Aldolase, fructose-bisphosphate aldolase; DHAP, dihydroxyacetone phosphate; FBP, fructose 1,6-bisphosphate; GAP, glyceraldehyde 3-phosphate; np-GAPDH, non-phosphorylating glyceraldehyde-3-phosphate dehydrogenase; p-GAPDH, phosphorylating glyceraldehyde-3-phosphate dehydrogenase; PGK, phosphoglycerate kinase; P_i_, inorganic phosphate; RuBP, ribulose 1,5-bisphosphate; TPI, triose-phosphate isomerase.

In plants, two major ^13^C fractionation systems can be distinguished (Wieloch *et al.*, 2018). Diffusion–Rubisco fractionation accompanies CO_2_ diffusion from ambient air into chloroplasts and fixation by Rubisco (Figs 1, 2; Farquhar *et al.*, 1982). It affects all carbon positions of plant glucose equally due to the single-carbon addition from CO_2_ to ribulose 1,5-bisphosphate by Rubisco (Wieloch *et al.*, 2018). In contrast, post-Rubisco fractionation accompanies metabolic processes downstream of Rubisco and is position specific (Schmidt *et al.*, 2015). Deconvolution of these fractionation types requires the intramolecular approach.

Elucidating physiological origins of specific ^13^C signals determines their value for applications within the plant and Earth sciences. Here, we first identify an intramolecular ^13^C signal at tree-ring glucose C-4. Next, we assess the potential of all enzyme-catalysed reactions within carbon metabolism of tree-ring and leaf cells for signal introduction, and develop experimentally testable theories on the mechanisms introducing the ^13^C-4 signal. Lastly, we test our theories by ^13^C fractionation modelling.

## Materials and methods

### Data basis

Here, we reanalyse intramolecular ^13^C signals in glucose from an annually resolved *Pinus nigra* tree-ring series reported previously (Wieloch *et al.*, 2018). These signals cover the period 1961–1995 and are expressed in terms of intramolecular ^13^C discrimination, Δ _*i*′_, where *i* denotes glucose C–H positions (Wieloch *et al.*, 2018). In this notation, positive values denote discrimination against ^13^C. Triose phosphate cycling in tree-ring cells causes fractional equilibration of ^13^C between glucose C-1 and C-6, C-2 and C-5, and C-3 and C-4 (see SI 2 in Wieloch *et al.*, 2021, Preprint). The prime marks data corrected for heterotrophic triose phosphate cycling (Wieloch *et al.*, 2018).

### Carbon isotope fractionation model

Carbon isotope fractionation by glyceraldehyde-3-phosphate dehydrogenase (GAPDH) was modelled following published basic procedures (Hayes, 2002). In the cytosol of leaves, GAPDH reactions proceed from glyceraldehyde 3-phosphate (GAP) to glycolytic 3-phosphoglycerate (3PGA). Since this corresponds to the direction of net flux under most physiological conditions, it is called the forward reaction throughout the manuscript. All models assume an open system at steady state with the following layout. Photosynthetic GAP has two fates, leaf-cytosolic 3PGA or fructose 1,6-bisphosphate (FBP) metabolism, with the latter supplying tree-ring glucose synthesis (Fig. 1). GAP committed to FBP metabolism is denoted GAP′ (corresponds to tree-ring glucose C-4 to C-6). No distinction is made between GAP′ entering tree-ring glucose synthesis directly or indirectly via leaf starch. Glycolytic conversion of GAP to 3PGA via phosphorylating GAPDH (p-GAPDH) involves 1,3-bisphosphoglycerate (1,3BPGA) to 3PGA conversion by phosphoglycerate kinase (PGK). PGK has no primary carbon isotope effect and is assumed to not fractionate. Hence, ^13^C fractionation between 3PGA and GAP′ is given as


$$\begin{array}{l} R_{3PGA}=\frac{1}{\alpha }R_{{GAP}^{\prime }\;} \end{array}$$ (1)

where *R* denotes ^13^C/^12^C ratios, and α denotes ^13^C isotope effects of GAPDH. Isotope mass balance of the system is given as


$$\begin{array}{l} F_{GAP}=fF_{3PGA}+(1-f)F_{{GAP}^{\prime }\;} \end{array}$$ (2)

where *F* denotes fractional abundances [^13^C/(^12^C+ ^13^C)], and *f* denotes the 3PGA commitment. *F* and *R* relate to each other as


$$\begin{array}{l} F=\frac{R}{1+R} \end{array}$$ (3)

Substituting *F* for *R* in Equation 2 yields


$$\begin{array}{l} \frac{R_{GAP}}{1+R_{GAP}}=f\frac{R_{3PGA}}{1+R_{3PGA}}+(1-f)\frac{R_{{GAP}^{\prime }\;}}{1+R_{{GAP}^{\prime }\;}} \end{array}$$ (4)

Here, it is convenient to substitute the unknown *R*_GAP_ by the known ^13^C/^12^C ratio of the primary carbon isotope standard, *R*_VPDB_. Substituting *R*_3PGA_ or *R*_GAP′_ in Equation 4 based on Equation 1 then gives


$$\begin{array}{l} \frac{R_{VPDB}}{1+R_{VPDB}}=f\frac{R_{{GAP}^{\prime }\;}/\alpha }{1+R_{{GAP}^{\prime }\;}/\alpha }+(1-f)\frac{R_{{GAP}^{\prime }\;}}{1+R_{{GAP}^{\prime }\;}} \end{array}$$ (5)

and


$$\begin{array}{l} \frac{R_{VPDB}}{1+R_{VPDB}}=f\frac{R_{3PGA}}{1+R_{3PGA}}+(1-f)\frac{\alpha R_{3PGA}}{1+\alpha R_{3PGA}} \end{array}$$ (6)

Solving Equations 5 and 6 for *R*_GAP′_ and *R*_3PGA_, respectively, gives


$$\begin{array}{l} c=\frac{-ad-a+b+d-bd\pm Z}{2(a-1)} \end{array}$$ (7)

and


$$\begin{array}{l} e=\frac{-a-ad+b+d-bd\pm Z}{2(ad-d)} \end{array}$$ (8)

with


$$\begin{array}{l} Z=\sqrt{a^{2}d^{2}-2ad^{2}+b^{2}d^{2}+2abd^{2}-2bd^{2}+d^{2}-2a^{2}d+2ad-2b^{2}d+2bd+a^{2}+b^{2}-2ab} \end{array}$$

where *a* to *e* denote *R*_VPDB_/(1+*R*_VPDB_), *f*, *R*_GAP′_, α, and *R*_3PGA_, respectively. While there are two mathematically correct solutions (±*Z*), only one of them returns plausible fractionation values (–*Z*). Isotope effects are given as


$$\begin{array}{l} \alpha =\frac{g\alpha_{f_{\left(np\right)}}+(1-g)\alpha_{f_{\left(p\right)}}}{\alpha_{r_{\left(p\right)}}} \end{array}$$ (9)

where *g* is the fractional contribution of non-phosphorylating GAPDH (np-GAPDH) to catalysing the forward reaction (GAP to 3PGA), and α _f(np)_, α _f(p)_, and α _r(p)_ are ^13^C isotope effects of the np-GAPDH forward reaction (GAP to 3PGA), the p-GAPDH forward reaction (GAP to 1,3BPGA), and the p-GAPDH reverse reaction (1,3BPGA to GAP), respectively. Modelling results were expressed in terms of ^13^C discrimination in per mille as


$$\begin{array}{l} \Delta_{x}\;\;\;[\text{ \textbackslash{}textperthousand}]=\left(\frac{R_{VPDB}}{R_{x}}-1\right)⁎1000 \end{array}$$ (10)

where *x* denotes either GAP′ or 3PGA. Due to the substitution of the unknown *R*_GAP_ by *R*_VPDB_ (see above), Δ _GAP′_ develops from zero and Δ _3PGA_ develops towards zero; that is, the model exclusively returns the ^13^C discrimination of the system of interest. Leaf-cytosolic 3PGA is partly reimported into chloroplasts, and chloroplast metabolism can be expected to transfer part of the GAPDH fractionation signal to other glucose carbon positions (see SI 1 in Wieloch *et al.*, 2021, Preprint). However, these effects are small and were neglected here for reasons of simplicity.

### Model parameterisation

*R*_VPDB_ was set to 0.0112372 (IAEA, 1995). Conversion of hydrated GAP to 1,3BPGA via p-GAPDH has a kinetic isotope effect of 1.0125 and an equilibrium isotope effect of 0.995 at the carbon position corresponding to glucose C-4 *in vitro* (Canellas and Cleland, 1991). However, p-GAPDH uses free GAP as substrate (Canellas and Cleland, 1991). The equilibrium between hydrated and free GAP has an estimated isotope effect of 1.0028 (Canellas and Cleland, 1991). Thus, conversion of free GAP to 1,3BPGA by p-GAPDH has a kinetic isotope effect of α _f(p)_=1.0097 (=1.0125/1.0028) and an equilibrium isotope effect of 0.9922 (=0.995/1.0028). Furthermore, the reverse reaction (1,3BPGA to GAP) has a kinetic isotope effect of α _r(p)_=1.0176 [=(1.0125/1.0028)/(0.995/1.0028)] and an equilibrium isotope effect of 1.0078 [=1/(0.995/1.0028)]. At 25 °C, >95% of all GAP is hydrated (Canellas and Cleland, 1991). However, modelling does not consider the hydration–dehydration equilibrium since outgoing pathways use free GAP and together consume incoming GAP completely. This should preclude the manifestation of fractionation effects related to GAP hydration/dehydration. Currently, α _f(np)_ is unknown and there is no reason to believe that α _f(np)_ equals α _f(p)_ since np-GAPDH and p-GAPDH share no sequence homology and belong to different enzyme families (Habenicht *et al.*, 1994; Michels *et al.*, 1994). Therefore, α _f(np)_ was set to 1 in accordance with the null hypothesis. However, better characterization of the system requires determination of α _f(np)_. Both *f* and *g* were varied between 0 and 1.

## Results and discussion

### Tree-ring glucose C-4 exhibits a ^13^C signal reflecting processes downstream of Rubisco

Several observations related to the dataset of intramolecular ^13^C/^12^C ratios published previously (see the Materials and methods) point to a ^13^C signal at glucose C-4 introduced by processes downstream of Rubisco.

#### Observation 1.

**Fig. 3.**
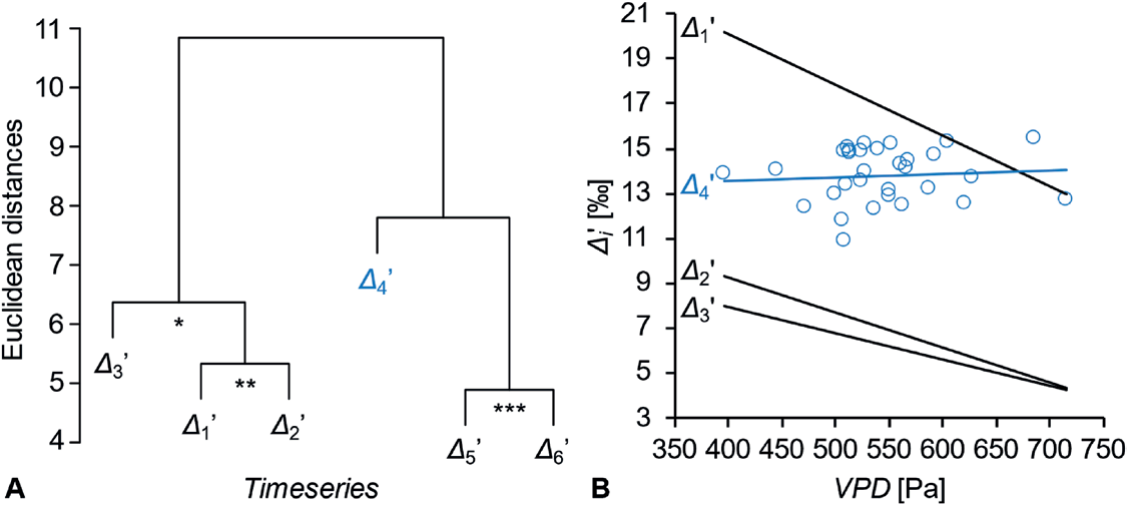
Hierarchical clustering of Δ _*i′*_ time series due to temporal co-variability. Members of clusters marked by asterisks are significantly correlated at the following significance levels: *P*≤0.05 (*), 0.01 (**), and 0.001 (***). (B) Relationships between Δ _*i*_ and growing season air vapour pressure deficit (VPD; March to November). Ordinary least squares regression models show significant negative relationships between VPD and Δ _1*′*_, Δ _2*′*_, and Δ _3*′*_ (Δ _1*′*_= –0.0226VPD+29.1, *R*^2^=0.46, *P*<0.0001; Δ _2*′*_= –0.0156VPD+15.5, *R*^2^=0.24, *P*<0.01; Δ _3*′*_= –0.0116VPD+12.6, *R*^2^=0.26, *P*<0.01) but not Δ _4*′*_ (Δ _4*′*_=0.0016VPD+12.9, *R*^2^=0.007, *P*>0.6). Δ _*i′*_ denotes time series of intramolecular ^13^C discrimination corrected for heterotrophic triose phosphate cycling (Wieloch *et al.*, 2018). Blue: time series discussed here. Data obtained from tree-ring glucose of *Pinus nigra* laid down from 1961 to 1995 at a dry site in the Vienna basin (*n*=31). To avoid cluttering, only Δ _4*′*_ data points are shown. Modified figure from Wieloch *et al.* (2018).

Hierarchical cluster analysis groups Δ _*i*′_ time series according to temporal co-variability. Time series that convey related information cluster together. Figure 3A shows association between Δ _4′_ and a cluster comprising Δ _5′_ and Δ _6′_. However, correlation between Δ _4′_ and the average of Δ _5′_ and Δ _6′_ is not significant (*r*=0.22, *P*>0.2, *n*=31). Thus, Δ _4′_ is the most independent time series among all Δ _*i*′_.

#### Observation 2.

All carbon in glucose enters metabolism via CO_2_ diffusion from ambient air into chloroplasts and fixation by Rubisco (Figs 1, 2). In the absence of post-Rubisco fractionation, all glucose carbon positions would exhibit equal ^13^C signals reflecting diffusion–Rubisco fractionation. In response to drought, isohydric plant species such as *Pinus nigra* close their stomata (Sade *et al.*, 2012; Roman *et al.*, 2015). This impedes CO_2_ diffusion and can result in low CO_2_ concentrations in chloroplasts, *C*_c_. With decreasing *C*_c_, Δ _*i*′_ decreases due to decreased ^13^C discrimination by the diffusion–Rubisco interface (Farquhar *et al.*, 1982). Figure 3B shows ordinary least squares regression models for relationships between Δ _*i*′_ and air vapour pressure deficit (VPD), a measure of air dryness. In line with expectations related to diffusion–Rubisco fractionation, VPD explains significant fractions of variation in Δ _1′_ to Δ _3′_ (*R*^2^>0.23, *P*<0.01, *n*=31, negative correlations). In contrast, Δ _4′_ and VPD are not related (*R*^2^=0.007, *P*>0.6, *n*=31; Wieloch *et al.*, 2018). This points to a fractionating metabolic process located downstream of Rubisco which removes the VPD-dependent diffusion–Rubisco signal from glucose C-4. Thus, post-Rubisco fractionation causes Δ _4′_ increases (^13^C-4 decreases) in response to increasing VPD.

#### Observation 3.

Among all Δ _*i*′_ time series, Δ _4′_ exhibits the lowest degree of explainable variance (±0.45‰ versus ±1.04‰ to ±3.37‰; Wieloch *et al.*, 2018). This corroborates the idea of a post-Rubisco fractionation effect annihilating the diffusion–Rubisco signal at glucose C-4.

### Elucidating the metabolic origin of the signal at glucose C-4: relevant processes

The VPD-dependent diffusion–Rubisco signal is present at glucose C-1 to C-3, yet positively correlated post-Rubisco fractionation processes may have overlapping effects at C-1 and C-2 (Fig. 3B; Wieloch *et al.*, 2018). In the following, an uncompromised diffusion–Rubisco signal is assumed to be preserved at glucose C-3. Relationships between growing season VPD and ^13^C discrimination have slopes of –0.0116‰ Pa^–1^ at glucose C-3 and 0.0016‰ Pa^–1^ at glucose C-4 (Fig. 3B). Hence, the relationship between VPD and post-Rubisco discrimination at glucose C-4 has an estimated slope of 0.0132‰ Pa^–1^. During the study period, VPD ranged from 396 Pa to 716 Pa. Thus, annihilation of the diffusion–Rubisco signal requires a post-Rubisco discrimination process with a range of 4.2‰ [=0.0132‰ Pa^–1^)×(716 Pa–396 Pa)].

Isotope fractionation signals are functions of observed isotope effects and relative carbon allocation into competing biochemical pathways (see fractionation modelling below; Hayes, 2002). Secondary carbon isotope effects (occurring at atoms with unaltered binding after chemical reactions due to indirect involvement in reaction mechanisms) are usually small and highly unlikely to cause a 4.2‰ fractionation effect. Therefore, the following discussion only considers primary carbon isotope effects (occur at atoms with altered binding after chemical reactions). In addition, a 4.2‰ fractionation effect in tree-ring glucose requires significant carbon allocation into pathways branching off from the glucose biosynthesis pathway. Therefore, the following discussion only considers reactions within central carbon metabolism of C_3_ plants. This includes the Calvin–Benson cycle, photorespiration, starch and sucrose synthesis and degradation, cellulose synthesis, the pentose phosphate pathway, glycolysis, and carbon metabolism downstream of phospho*enol*pyruvate (PEP). Carbon allocation into other pathways is presumably small, especially when integrated over the course of growing seasons, the time frame of tree-ring formation. Thus, these processes are highly unlikely to introduce 4.2‰ fractionation effects, and our chosen restrictions should not lead to incorrect conclusions.

In addition, it is important to note that the post-Rubisco signal at glucose C-4 is introduced at the level of three-carbon compounds. Reactions at other levels do not modify carbon bonds that become glucose C-4. Signals in three-carbon compounds can enter glucose metabolism via dihydroxyacetone phosphate (DHAP; glucose C-1 to C-3) and GAP (glucose C-4 to C-6; Figs 1, 2).

### Exclusion of metabolic locations as the origin of the Δ_4′_ signal

Based on current knowledge about plant metabolism, we can exclude several locations as the origin of the post-Rubisco signal at glucose C-4.

#### Exclusion 1.

In tree-ring cells, GAP and DHAP are in equilibrium (Wieloch *et al.*, 2021, Preprint). This can be expected to cause ^13^C signal equilibration between GAP and DHAP carbon positions corresponding to glucose C-4 and C-3, respectively (Figs 1, 2). However, the post-Rubisco signal at glucose C-4 is clearly confined to this position (Fig. 3). This excludes tree-ring cells as the signal origin.

#### Exclusion 2.

Metabolism feeding or consuming the GAP pool in chloroplasts, such as the Calvin–Benson cycle, photorespiration, and the non-mevalonate pathway, can be excluded as the signal origin for the following reason. Glucose synthesis involves the conversion of GAP (C-4 to C-6) to DHAP (C-3 to C-1; Figs 1, 2). This entails ^13^C signal propagation from the GAP carbon position corresponding to glucose C-4 to the DHAP carbon position corresponding to glucose C-3. However, the discussed signal is confined to glucose C-4 (Fig. 3).

#### Exclusion 3.

PEP feeds (*inter alia*) into anaplerotic carbon fixation, the shikimate pathway, the non-mevalonate pathway, nitrogen assimilation, and mitochondrial metabolism. In mesophyll cells under normal growth, carbon flux from these processes back to PEP is negligible (Wieloch *et al.*, 2021, Preprint). Thus, processes located downstream of PEP cannot feed significant amounts of carbon and associated ^13^C signals into glucose metabolism. This excludes them as the signal origin.

#### Exclusion 4.

Reactions of the oxidative part of the pentose phosphate pathway and reactions leading from FBP to starch, sucrose, and tree-ring cellulose do not modify carbon positions that become glucose C-4. This excludes these pathways as the signal origin.

#### Exclusion 5.

It is generally accepted that the non-oxidative part of the pentose phosphate pathway in the cytosol of leaves is incomplete. Specifically, *Arabidopsis thaliana* lacks genes encoding cytosolic isoforms of transketolase (EC 2.2.1.1) and transaldolase (EC 2.2.1.2; Kruger and von Schaewen, 2003). To our knowledge, these genes have not been found in other plant species. Since transketolase and transaldolase catalyse the only reactions of the pentose phosphate pathway modifying three-carbon molecules, we can exclude this part of metabolism as the signal origin.

#### Exclusion 6.

Triose-phosphate isomerase (TPI) catalyses interconversions between the DHAP hydroxyl carbon (glucose C-3) and the GAP aldehyde carbon (glucose C-4; Figs 1, 2). Due to mass balance, any ^13^C effect by TPI at one of these carbon positions will have an equally sized inverse effect at the other carbon position. However, while the VPD-dependent diffusion–Rubisco signal is absent at glucose C-4, it is not enhanced at glucose C-3 (Fig. 3B). This excludes TPI as the signal origin.

#### Exclusion 7.

A similar exclusion criterion applies to fructose-bisphosphate aldolase (aldolase) which catalyses the condensation of DHAP and GAP to FBP (Figs 1, 2). At chemical equilibrium, aldolase causes similarly sized ^13^C enrichments at FBP carbon positions corresponding to glucose C-3 and C-4 (Table 1; Gleixner and Schmidt, 1997). With shifts towards kinetic conditions (towards FBP synthesis), these effects develop in opposite directions. However, the magnitude of the C-3 shift (15.9‰) distinctly exceeds the magnitude of the C-4 shift (–3.2‰). Thus, a shift from equilibrium to kinetic conditions which removes the VPD-dependent diffusion–Rubisco signal from C-4, should cause a 5-fold increase of this signal at C-3. However, this is not supported by the data (Fig. 3B).

**Table 1.** *In vitro* ^13^C isotope effects of aldolase and p-GAPDH (Canellas and Cleland, 1991; Gleixner and Schmidt, 1997)

Aldolase							p-GAPDH		
	FBP			DHAP	+	GAP	GAP		1,3BPGA
**C-3**		**C-4**		**C-3**		**C-4**	**C-4**		**C-4**
			**→**	1.0159		0.9968		**→**	1.0097
0.9964		0.9951	**↔**	1.0036		1.0049	1.0078	**↔**	0.9922
1.0123		0.9919	**←**				1.0176	**←**	

→ Kinetic isotope effect, ↔ equilibrium isotope effect. Carbon numbering corresponds to related carbon positions in glucose. Reported values are in italics. Calculated values are not italicized. Effects of p-GAPDH refer to free GAP, not hydrated GAP.

### GAPDH in the cytosol of leaves introduces the signal at glucose C-4

After excluding several metabolic locations as the origin of the signal at glucose C-4, glycolysis in the cytosol of leaves is left for consideration. In this pathway, p-GAPDH and np-GAPDH modify the GAP carbon position corresponding to glucose C-4 (Figs 1, 2). Thus, GAPDH in the cytosol of leaves is the most plausible origin of the post-Rubisco signal at glucose C-4.

Leaf-cytosolic GAP is a precursor of glycolytic 3PGA and FBP, a precursor of tree-ring glucose (Fig. 1). Both pathways can be expected to carry substantial flux (Wieloch *et al.*, 2021, Preprint). Thus, GAP constitutes a major branch point in carbon metabolism principally enabling the manifestation of ^13^C effects. However, introduction of uncorrelated ^13^C signals at glucose C-4 and C-3 as observed (Fig. 3) requires a lack of isotopic equilibrium between GAP and DHAP in the cytosol of leaves. Previously, we provided evidence and arguments in support of this lack (Wieloch *et al.*, 2021, Preprint).

### Ecophysiological mechanisms introduce the signal at glucose C-4

Above, we propose leaf-cytosolic GAPDH as the most plausible origin of the post-Rubisco ^13^C signal at glucose C-4. To our knowledge, ^13^C isotope effects of np-GAPDH have not been reported. In contrast, conversion of free GAP to 1,3BPGA by p-GAPDH has a kinetic isotope effect of 1.0097 and an equilibrium isotope effect of 0.9922 at the carbon position corresponding to glucose C-4 *in vitro* (Table 1; Canellas and Cleland, 1991). To test whether these effects can remove the VPD-dependent diffusion–Rubisco signal, we modelled ^13^C discrimination pertaining to two conceivable flux change scenarios around GAPDH.

#### Scenario 1: Varying carbon commitment to 3PGA versus FBP metabolism.

Reported concentrations of GAP and 3PGA in suspension-cultured cells of *Catharanthus roseus* correspond to equilibrium conditions around p-GAPDH and PGK (Kubota and Ashihara, 1990). Hence, the first scenario assumes (i) inactivity of np-GAPDH; (ii) equilibrium between GAP and 3PGA catalysed by p-GAPDH and PGK; and (iii) varying relative carbon commitment to leaf-cytosolic 3PGA versus FBP metabolism, *f*.

**Fig. 4.**
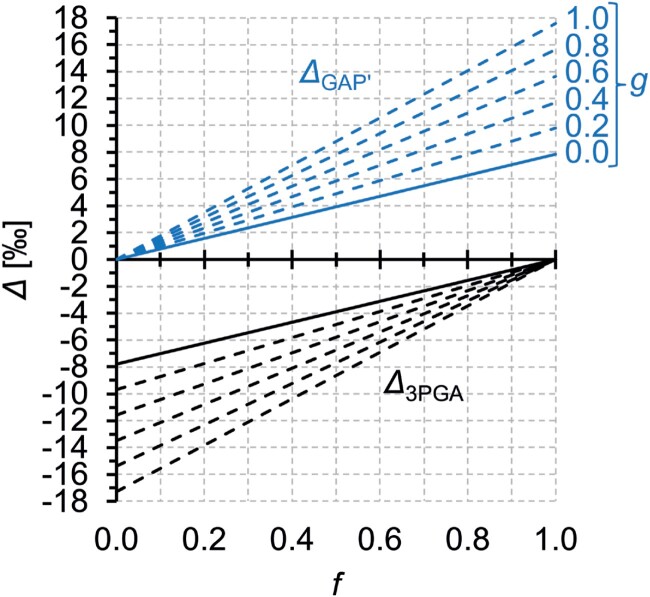
Modelled ^13^C discrimination, Δ, by leaf-cytosolic glyceraldehyde-3-phosphate dehydrogenases (GAPDHs) at carbon positions corresponding to tree-ring glucose C-4. In this model, cytosolic glyceraldehyde 3-phosphate (GAP) has two fates: (i) glycolytic 3-phosphoglycerate (3PGA) and downstream metabolism, and (ii) fructose 1,6-bisphosphate (FBP) and downstream metabolism including the biosynthesis of tree-ring glucose (Fig. 1). Commitment of GAP to 3PGA versus FBP metabolism is given on the abscissa as *f* (e.g. *f*=0: all GAP enters FBP metabolism, *f*=1: all GAP enters 3PGA metabolism). Leaf cytosol contains two entirely unrelated GAPDHs (no sequence homology). While the irreversible non-phosphorylating (np-) GAPDH can catalyse GAP to 3PGA forward reactions only, the reversible phosphorylating (p-) GAPDH together with phosphoglycerate kinase can catalyse both the forward and reverse reaction. The contribution of np- versus p-GAPDH to catalysing the GAP to 3PGA forward reaction is given as *g* (e.g. *g*=0, forward reaction catalysed exclusively by p-GAPDH; *g*=1, forward reaction catalysed exclusively by np-GAPDH). GAP entering the system has Δ=0. Blue lines, discrimination in GAP committed to FBP metabolism denoted Δ _GAP*′*_; black lines, discrimination in 3PGA denoted Δ _3PGA_; solid lines, discrimination without activity of np-GAPDH (*g*=0); dashed lines, discrimination when np-GAPDH catalyses fractions of the GAP to 3PGA forward reaction. p-GAPDH has a kinetic isotope effect of 1.0097 and an equilibrium isotope effect of 0.992 *in vitro* (Table 1; Canellas and Cleland, 1991). In contrast, the ^13^C isotope effect of np-GAPDH is unknown and, therefore, is assumed to be 1 in accordance with the null hypothesis.

Figure 4 shows ^13^C discriminations modelled for this scenario. Increasing *f* increases the ^13^C discrimination in remaining GAP, namely GAP entering FBP metabolism (denoted Δ _GAP′_, solid blue line) relative to GAP entering the system (Δ _GAP_=0). A discrimination range of 4.2‰ would require *f* to vary with a range of ~0.535. While significant changes in relative carbon commitment to 3PGA versus FBP metabolism in response to environmental variability can be expected (Wieloch *et al.*, 2021, Preprint), leaf-level carbon commitment to 3PGA metabolism >0.535 over an entire growing season is unlikely. Nevertheless, varying *f* may contribute to the post-Rubisco signal at glucose C-4 and may respond to VPD as follows.

With increasing VPD, *C*_c_ decreases (see above). This may result in decreased net carbon assimilation and flux into FBP metabolism, while flux into 3PGA metabolism is maintained. For instance, under drought, PEP carboxylase (EC 4.1.1.31, located downstream of 3PGA) is up-regulated while carbon assimilation by the Calvin–Benson cycle can be expected to decrease (O’Leary *et al.*, 2011).

#### Scenario 2: Varying contribution of np-GAPDH versus p-GAPDH to catalysing the GAP to 3PGA forward reaction.

Numerous observations suggest a significant contribution of np-GAPDH to catalysing the GAP to 3PGA forward reaction of glycolysis (Fig. 1; Wieloch, 2021). Hence, the second scenario assumes (i) activity of both np-GAPDH catalysing the forward reaction (GAP to 3PGA), and p-GAPDH and PGK catalysing forward and reverse reactions (Fig. 1); (ii) np-GAPDH has no ^13^C isotope effect; (iii) apparent equilibrium between GAP and 3PGA with respect to p-GAPDH and PGK; (iv) varying relative carbon commitment to 3PGA versus FBP metabolism, *f*; and (v) varying relative contribution of np-GAPDH versus p-GAPDH to catalysing the GAP to 3PGA forward reaction, *g*.

Figure 4 shows ^13^C discriminations modelled for this scenario. Increasing *g* increases the ^13^C discrimination in remaining GAP (denoted Δ _GAP′_, dashed blue lines) relative to *g*=0 (solid blue line, scenario 1). Simultaneously, the slope of the relationship between ^13^C discrimination and *f* increases. Combined action of these effects may result in the required discrimination range of 4.2‰. For instance, a given growing season may exhibit *f*=*g*=0.1 corresponding to Δ _GAP′_=0.88 (Fig. 4). The growing season at the other end of the range with Δ _GAP′_=5.08 may then exhibit, for example, *g*=0.8 and *f*=0.325 (Fig. 4). This corresponds to flux changes of Δ*g*=0.7 and Δ*f*=0.225 which may be physiologically feasible. Thus, varying *g* may contribute to the post-Rubisco signal at glucose C-4 and may respond to VPD as follows.

With increasing VPD, *C*_c_ decreases (see above). Decreasing *C*_c_ promotes an energy excess in chloroplasts due to decreasing ATP and NADPH demands in the Calvin–Benson cycle while electron input by the light reactions is maintained (Huner *et al.*, 1996; Wilhelm and Selmar, 2011; Vanlerberghe *et al.*, 2015). Excess energy promotes the synthesis of reactive oxygen species especially superoxide and H_2_O_2_. Simultaneously increased photorespiration adds to the oxidative load via peroxisomal synthesis of H_2_O_2_ by glycolate oxidase (EC 1.1.3.15; Noctor *et al.*, 2002; Cruz de Carvalho, 2008). In the cytosol, oxidative conditions lead to increased NADPH consumption by antioxidant systems. As NADPH decreases, np-GAPDH can be expected to become increasingly active (Kelly and Gibbs, 1973; Iglesias and Losada, 1988; Scagliarini *et al.*, 1990; Gómez Casati *et al.*, 2000). Furthermore, p-GAPDH is 63 times more susceptible to oxidative inhibition (especially by H_2_O_2_) than np-GAPDH (Piattoni *et al.*, 2013). Thus, the relative contribution of np-GAPDH versus p-GAPDH to catalysing the GAP to 3PGA forward reaction, *g*, can be expected to increase with an increasing oxidative load due to increasing VPD.

Both scenarios result in positive correlation between VPD and ^13^C discrimination at glucose C-4 and can thus annihilate the diffusion–Rubisco signal at this position.

### What information does the post-Rubisco ^13^C signal at glucose C-4 convey?

According to theory and modelling above, the post-Rubisco signal at glucose C-4 contains two kinds of (potentially convoluted) information. First, it reports relative carbon commitment to leaf-cytosolic 3PGA versus FBP metabolism, *f* (scenario 1). Second, it reports relative contributions of np-GAPDH versus p-GAPDH to catalysing the GAP to 3PGA forward reaction of glycolysis, *g* (scenario 2).

At *g*=1, np-GAPDH catalyses all forward reactions (GAP to 3PGA) while p-GAPDH together with PGK catalyses all reverse reactions (3PGA to GAP). In a recent Viewpoint, this flux mode was proposed based on differences in biochemical properties of np- and p-GAPDH, and was termed the cytosolic oxidation–reduction (COR) cycle (Wieloch, 2021). COR cycling may constitute a central hub in leaf-cytosolic energy metabolism, with np-GAPDH supplying NADPH in the forward reaction (GAP to 3PGA) and PGK and p-GAPDH consuming ATP and NADH in the reverse reactions (3PGA to 1,3BPGA to GAP; Fig. 2). The idea of COR cycling is supported by the fact that scenario 1 can probably not account for the entire variability of the post-Rubisco signal at glucose C-4 (see above). Furthermore, positive correlation between VPD and ^13^C discrimination related to COR cycling makes sense in the context of plant homeostasis (Fig. 3). With increasing VPD, *g* increases due to an increasing oxidative load caused by photorespiration and excess energy (see above). Concomitantly increasing NADPH supply by np-GAPDH can fuel antioxidant systems, while the reverse reactions consume excess energy. Thus, increasing COR cycling in response to increasing VPD may help to maintain the leaf-cytosolic redox balance.

In addition to reporting physiological changes, the post-Rubisco signal at glucose C-4 conveys information about underlying environmental and/or developmental cofactors. Overall, analysis of the reported ^13^C signal may enable an improved understanding of leaf carbon and energy metabolism.

## Data Availability

All data supporting the findings of this study are available within the paper.

## Abbreviations

Aldolase: fructose-bisphosphate aldolase (EC 4.1.2.13)
1,3BPGA: 1,3-bisphosphoglycerate
COR: cytosolic oxidation–reduction
DHAP: dihydroxyacetone phosphate
FBP: fructose 1,6-bisphosphate
GAP: glyceraldehyde 3-phosphate
np-GAPDH: non-phosphorylating glyceraldehyde-3-phosphate dehydrogenase (EC 1.2.1.9)
PEP: phosphoenolpyruvate
3PGA: 3-phosphoglycerate
p-GAPDH: phosphorylating glyceraldehyde-3-phosphate dehydrogenase (EC 1.2.1.12)
PGK: phosphoglycerate kinase (EC 2.7.2.3)
TPI: triose-phosphate isomerase (EC 5.3.1.1)
VPD: air vapour pressure deficit
Δ*i′*: intramolecular ^13^C discrimination corrected for heterotrophic triose phosphate cycling, with *i* denoting the glucose carbon position.
